# Editorial: Cyanobacterial biology in twenty-first century

**DOI:** 10.3389/fmicb.2023.1184669

**Published:** 2023-03-29

**Authors:** Prashant Kumar Singh, Vijay Pratap Singh, Michel Rodrigo Zambrano Passarini, Ajay Kumar

**Affiliations:** ^1^Department of Biotechnology, Mizoram University, Pacchunga University College, Aizawl, India; ^2^Department of Botany, CMP Post Graduate College, University of Allahabad, Allahabad, India; ^3^Latin American Institute of Life and Nature Sciences (ILACVN), Environmental Biotechnology Laboratory, Federal University of Latin American Integration (UNILA), Foz do Iguaçu, Paraná, Brazil; ^4^Department of Post-Harvest, Agriculture Research Organization, The Volcani Centre, Rishon LeZion, Israel

**Keywords:** cyanobacteria, bioremediation, microcystin (MC), stress, CRISPR

*Cyanobacterial Biology in the twenty-first century* Research Topic gathers different contributions high- lighting the cyanobacterial basic to advanced research going worldwide. Cyanobacteria are ancient photosynthetic prokaryotes and sustained during evolution. This sustainability is because of their fitness (stress adaptations) to the extreme environment and ability to fix atmospheric carbon dioxide and nitrogen. These tiny creatures have recently gained popularity due to their ability to produce biodegradable plastics (PHAs; polyhydroxyalkanoates) and biofuel; carbon sequestration ability, and they are a natural reservoir of various metabolites (alkaloids, ribosomal peptides, non-ribosomal peptides, cyanotoxins, and polyketides), food additives and drug designing. Furthermore, their universal presence, rapid growth, survivability under extreme conditions and minimum growth requirements make them a popular resource for sustainable agriculture. The cyanobacterial photosynthetic lamellae (thylakoid membranes) possess substantial amounts of lipids and offer greater photosynthesis efficiency; hence these attributes could be exploited to produce biofuel and bioplastic. The production of bioplastics uses the carbon sink concept and cyanobacteria's role in PHAs (polyhydroxyalkanoates) production as a source of intracellular energy. It is warranted to mention that cyanobacteria are a great source of biologically active compounds with anticancer, antifungal, antibacterial and antiviral properties. However, the knowledge behind the mode of action associated with these active compounds is still limited, even with advancements in the latest omics and technologies. Henceforth, this Research Topic mainly aims to assemble the most recent global cyanobacterial research to comprehend cyanobacteria's metabolism and physiology for better utilization for human welfare. In the current issue, nine articles have been published, which empowers our understanding of cyanobacteria's role in managing abiotic and biotic stress, biostimulants, biofuel production, and molecular aspects associated with cyanobacterial physiology.

Agarwal et al. emphasize different cyanobacterial species' ability to produce biodegradable plastics and their significance as green and clean energy sources. Future generations can rely on cyanobacteria to reach sustainable environmental goals due to their role in producing (PHAs) polyhydroxyalkanoates as a better alternative to conventional plastics. Compared to other energy sources, such as green plants, Cyanobacteria shows a more effective photosynthetic mechanism, which provides maximum output with limited affordable land resources. An unconventional solution for a sustainable future is the synthesis of biodiesel from cyanobacteria, which reduces the emission of hazardous sulfur and prevents the addition of aromatic hydrocarbons with a high oxygen content and excellent combustion potential.

Ding et al. investigated the molecular aspect of cyanobacterial physiology—two accessory CARF-domain proteins from cyanobacterium *Synechocystis* sp. PCC III-D type CRISPR systems have been isolated and characterized. They found a model organism, *Synechocystis* sp. PCC 6803 contains ring nuclease proteins and a functional CARF-domain effector. SyCsx1, a cyclic tetraadenylate (cA4)-dependent RNase with strong specificity for cytosine nucleotides, is the homolog of Csx1. SyCsm6, a second CARF-domain protein that shares similarities with Csm6 effectors, broke down cOAs while attenuating SyCsx1 RNase activity. Their findings imply that *Synechocystis* CRISPR systems provide a multilayered cA4-mediated defense mechanism.

He et al. demonstrate *Prochlorococcus* global gene expression changes due to low salinity stress and their expanding salinity range. In this investigation, researchers discovered that *Prochlorococcus* strain NATL1A, which is low-light adapted, and strain MED4, which is high-light adapted, could acclimate in the lowest salinities of 25 and 28 psu. While both strains were grown in salinity more bass than 34 psu, the effective quantum yield of PSII photochemistry (Fv/Fm) showed that both the strains were stressed. To adapt to low salinity, the transcript of genes involved in translation, biogenesis, ribosomal structure and ATP generation was downregulated by NATL1A, whereas MED4 upregulated genes linked to photosynthesis. Moreover, the iron ABC transporter gene idiA was upregulated in both strains, indicating that low salinity acclimated cells may be iron deficient.

Kar et al. reviewed the characteristics, potential uses, and critical analysis of cyanobacterial metabolites and the mechanisms of action associated with contributing to the hunt for novel antimicrobials. Singh K.B. et al.  addressed the limitations of using tetrapyrrole rings and natural nutrient supplements as therapeutic agents. Also, they demonstrated the various aspects of tetrapyrrole rings in the food and pharmaceutical industries. Singh R.P. et al. isolated several microalgae species from the wastewater environment, and their lipid accumulation and maximum growth rates were assessed. All isolated microalgae can adapt to a carbon source evaluated by adding sodium bicarbonate to BG-11N+ media. Further best Microalgal strains were chosen for biofuel feedstock based on growth parameters and sodium bicarbonate uptake rates among all the selected strains *Scenedesmus* sp. BHU1 strain was found to be highly efficient for biofuel production.

Srivastava et al. perform research to understand the information gaps about how pretreatment affects cyanobacteria. It is analyzed that the detrimental effects of salt can be reduced in filamentous cyanobacteria after pretreatment with heat, hence providing a basis for increased cyanobacterial tolerance to salt stress. Yalcin et al. examined the effects of kanamycin, ampicillin, cefotaxime and tetracycline on photosynthetic capacity and pigment fluorescence in *Fremyella diplosiphon* strains. Studies on the hormetic effect of antibiotics on *F. diplosiphon* show that excessive antibiotic concentrations significantly damage cellular functionality, and appropriate antibiotic doses stimulate cellular growth. Finally, Guo et al. identified a new microcystinase (MlrC) enzyme that degrades microcystin toxins produced by cyanobacteria.

It can be concluded that cyanobacteria show wide adaptability to biotechnological uses. They have been employed in drug development, bioremediation and medical diagnostics. In addition, they are potent sources of bioplastics, bioactive substances, food supplements, biofertilizers and energy. To enable their use, the additional study must concentrate on the thriving axenic culture of these microorganisms. In addition, new techniques must be developed to allow the cultivation of previously “uncultivable” strains.

We hope that the reader will find in this Research Topic a valuable reference for the state-of-the-art in the emerging field of tools rooted in information theory and applied to neuroscience.

## Author contributions

All authors listed have made a substantial, direct, and intellectual contribution to the work and approved it for publication.

